# Efficacy and safety of occipital nerve blocks in cluster headache: a prospective observational study

**DOI:** 10.1186/1129-2377-14-S1-P60

**Published:** 2013-02-21

**Authors:** C Gaul, J Roguski, H Shanib, A Totzeck, K Görlinger, HC Diener, R Weber

**Affiliations:** 1 Migraine- and Headache Clinic Königstein, Germany; 2 Headache Center Universityhoispital Essen, Germany; 3 University Hospital Essen, UK; 4 University Hospital Essen, Germany

## Background

Cluster headache (CH) is characterized by severe trigemino-autonomic cephalagias. Treatment of CH consists of acute attack aborting and prophylactic strategies. However, a substantial number of patients do not have sufficient control of CH attacks despite oxygen inhalation, triptans, oral steroids and verapamil. To date only two small randomized trial and retrospective case series have investigated the efficacy and safety of occipital nerve blocks in CH.

## Methods

The effect of a single infiltration of the ipsilateral greater and lesser occipital nerve using a long-acting corticosteroid (10 mg triamcinolone) and anaesthetic (Bupivacaine 0.5%) was prospectively investigated in 101 CH patients (61 episodic CH, 40 chronic CH) who did not have sufficient control of CH attacks in a tertiary headache center during July 2010 and November 2011. Attack frequency, pain intensity and side effects were recorded by repetitive standardized interviews at days 3 and 7 after infiltration and thereafter weakly until reoccurrence of attacks.

## Results

The mean attack frequency was 2.9 ± 2.5 (eCH) and 3.3 ± 2.9 (cCH) at baseline. This was reduced to 2.2 ± 1.7 (eCH) respective 2.5 ± 2.3 (cCH) after 7 days. 67.2% (eCH) and 50% (cCH) became attack free. 10.9% of the patients reported at least one side effect. Most frequent side effects were: nausea (0.9%), pressure (0.9%) or pain (1.8%) at the injection site, tension type headache (4.6%) and retroorbital pain (1.8%). 83% of patients would repeat nerve block treatment.

## Conclusion

Occipital nerve block is an easy, safe and effective treatment option for exacerbation of eCH and cCH which can suppress attacks temporarily in a high number of patients with eCH and cCH and results in a complete response in a substantial number of patients with eCH.
